# Extreme pollution reshapes microbial community assembly in a tropical urban river

**DOI:** 10.1093/femsec/fiag083

**Published:** 2026-07-29

**Authors:** Luiz Ricardo Olchanheski, Felipe Rezende de Lima, Lina Rocío del Pilar Rada Martinez, Eliane Gonçalves da Silva, Mabel Patricia Ortiz-Vera, Maria Inês Zanoli Sato, Gabriel Padilla, Welington Luiz Araújo

**Affiliations:** LABMEM/NAP-BIOP, Department of Microbiology, Institute of Biomedical Sciences, University of São Paulo, 1374 -Ed. Biomédicas II, Cidade Universitária, 05508-900, São Paulo, SP, Brazil; Department of Basic Health Sciences, State University of Maringá, Av. Colombo, 5790 – Zona 7, 87020-900, Maringá, PR, Brazil; LABMEM/NAP-BIOP, Department of Microbiology, Institute of Biomedical Sciences, University of São Paulo, 1374 -Ed. Biomédicas II, Cidade Universitária, 05508-900, São Paulo, SP, Brazil; LABMEM/NAP-BIOP, Department of Microbiology, Institute of Biomedical Sciences, University of São Paulo, 1374 -Ed. Biomédicas II, Cidade Universitária, 05508-900, São Paulo, SP, Brazil; LABMEM/NAP-BIOP, Department of Microbiology, Institute of Biomedical Sciences, University of São Paulo, 1374 -Ed. Biomédicas II, Cidade Universitária, 05508-900, São Paulo, SP, Brazil; LABMEM/NAP-BIOP, Department of Microbiology, Institute of Biomedical Sciences, University of São Paulo, 1374 -Ed. Biomédicas II, Cidade Universitária, 05508-900, São Paulo, SP, Brazil; Department of Environmental Analysis, Environmental Company of São Paulo State (CETESB), Av. Prof. Frederico Hermann Jr., 345, 05459-900, São Paulo, SP, Brazil; LABMEM/NAP-BIOP, Department of Microbiology, Institute of Biomedical Sciences, University of São Paulo, 1374 -Ed. Biomédicas II, Cidade Universitária, 05508-900, São Paulo, SP, Brazil; LABMEM/NAP-BIOP, Department of Microbiology, Institute of Biomedical Sciences, University of São Paulo, 1374 -Ed. Biomédicas II, Cidade Universitária, 05508-900, São Paulo, SP, Brazil

**Keywords:** microbial community assembly, anthropogenic disturbance, ecological drift, pelagic bacterial communities, Water Quality Index gradient, tropical urban rivers

## Abstract

Anthropogenic pollution profoundly alters urban rivers, yet its effects on microbial community assembly remain poorly understood. Here, we investigated spatial patterns of bacterial community structure and assembly along the Tietê River (Brazil), a heavily polluted tropical urban river. We combined 16S rRNA gene amplicon sequencing with physicochemical characterization across a Water Quality Index (WQI) gradient and quantified ecological assembly processes using the iCAMP framework. Contrary to the expected pollution-driven loss of diversity, alpha diversity was significantly higher in severely degraded stretches. Community composition shifted from freshwater-associated taxa, such as Comamonadaceae, in less impacted sites to pollution-tolerant groups, particularly Arcobacteraceae, in degraded reaches. Homogeneous selection predominated in Good and Regular WQI sites but declined markedly under Poor WQI conditions, where drift and dispersal limitation became the dominant assembly processes. These results indicate that extreme pollution alters the nature of environmental filtering, favoring disturbance-tolerant functional traits distributed across phylogenetically diverse taxa and increasing the influence of stochastic assembly. Consequently, taxonomic composition becomes less predictable from local environmental conditions. Our findings provide a mechanistic framework for understanding how chronic anthropogenic disturbance reshapes microbial community assembly in tropical urban rivers.

## Teaser

Microbial community in a polluted tropical river.

## Introduction

The escalating intensity of anthropogenic activities poses a significant and globally disseminated threat to the ecological integrity of aquatic ecosystems (Liu et al. 2023). In urban centers, these pressures are exacerbated by inadequate wastewater treatment and unplanned urbanization (Silva et al. 2024), leading to severe physicochemical alterations. Such alterations include water quality degradation from massive discharges of domestic sewage (Chedadi et al. 2023), agricultural runoff, and industrial effluents (Castro et al. 2021), which promote eutrophication and contamination by heavy metals, agrochemicals, and microplastics (Sun et al. 2022). Furthermore, hydromorphological interventions induce dynamic imbalances in river systems, resulting in channel simplification (Brenna et al. 2024).

The Tietê River, the most extensive fluvial system in the state of São Paulo, drains one of Brazil’s primary demographic and economic regions (Brenna et al. 2024, Moraes de et al. 2024). Despite its importance, the river suffers from severe environmental degradation due to the daily discharge of substantial domestic and industrial effluents (Mariano et al. 2025). This input leads to the accumulation of organic matter and toxic substances and creates persistent hypereutrophic condition, acting as a chemical barrier to the dispersal of aquatic organisms (Gonçalves et al. 2025). Aquatic microbial communities are fundamental biological elements that drive energy flow and biogeochemical cycling in freshwater ecosystems (Paula de et al. 2023). Indispensable to ecosystem health, micro-organisms perform vital functions, including biomass production, mineralization of essential elements, and bioremediation of xenobiotics compounds. In riverine sediments, bacterial communities are crucial for element cycling and serve as reliable bioindicators of environmental conditions (Zhang et al. 2021, Paula de et al. 2023). Due to their rapid response to selective pressures, microbial communities are considered an essential metric for assessing environmental health.

The intensification of anthropogenic activities has increased the inputs of emerging contaminants and excess nutrients into riverine systems (Gao et al. 2022). These disturbances can alter ecological integrity and biodiversity patterns, driving taxonomic and functional shifts in microbial communities (Liu et al. 2021, Zhang et al. 2021). Under such conditions, selective pressures may favor pollutant-tolerant taxa and specific metabolic pathways involved in contaminant transformation, potentially increasing functional redundancy within microbial assemblages (Liu et al. 2021). Accordingly, heavily impacted environments such as the Tietê River are hypothesized to exhibit reduced microbial diversity, which may be associated with simplified community structures and decreased stability, rendering them more susceptible to additional environmental stressors. In parallel, polluted aquatic ecosystems can favor the enrichment of antimicrobial resistance genes (ARGs) through co-selection mechanisms linked to toxic contaminants and antibiotic residues, with potential implications for microbial community functioning and broader One Health concerns (Regina et al. 2021).

Despite growing interest in these effects, a comprehensive understanding of how multiple anthropogenic stressors jointly shape the diversity, composition, and assembly processes of bacterial communities in a large tropical river system remains limited. Previous studies have highlighted the severe pollution of the Tietê River; however, the ecological mechanisms underlying microbial community dynamics, particularly the relative roles of deterministic and stochastic processes, have not been systematically examined at the watershed scale. In this study, we investigated spatial variation in bacterial communities along the Tietê River, integrating taxonomic and environmental data to infer community assembly mechanisms. We hypothesize that in heavily polluted river stretches, bacterial community assembly would be ruled by deterministic processes associated with strong environmental filtering, whereas in less impacted stretches, stochastic processes exert a greater influence. Specifically, our objectives were to (i) characterize taxonomic shifts in bacterial communities along a gradient of anthropogenic impact, (ii) identify key physicochemical variables associated with community structure, and (iii) quantify the relative contributions of deterministic and stochastic processes to bacterial community assembly.

## Material and methods

### Sampling sites and physicochemical parameters

**Figure 1:**
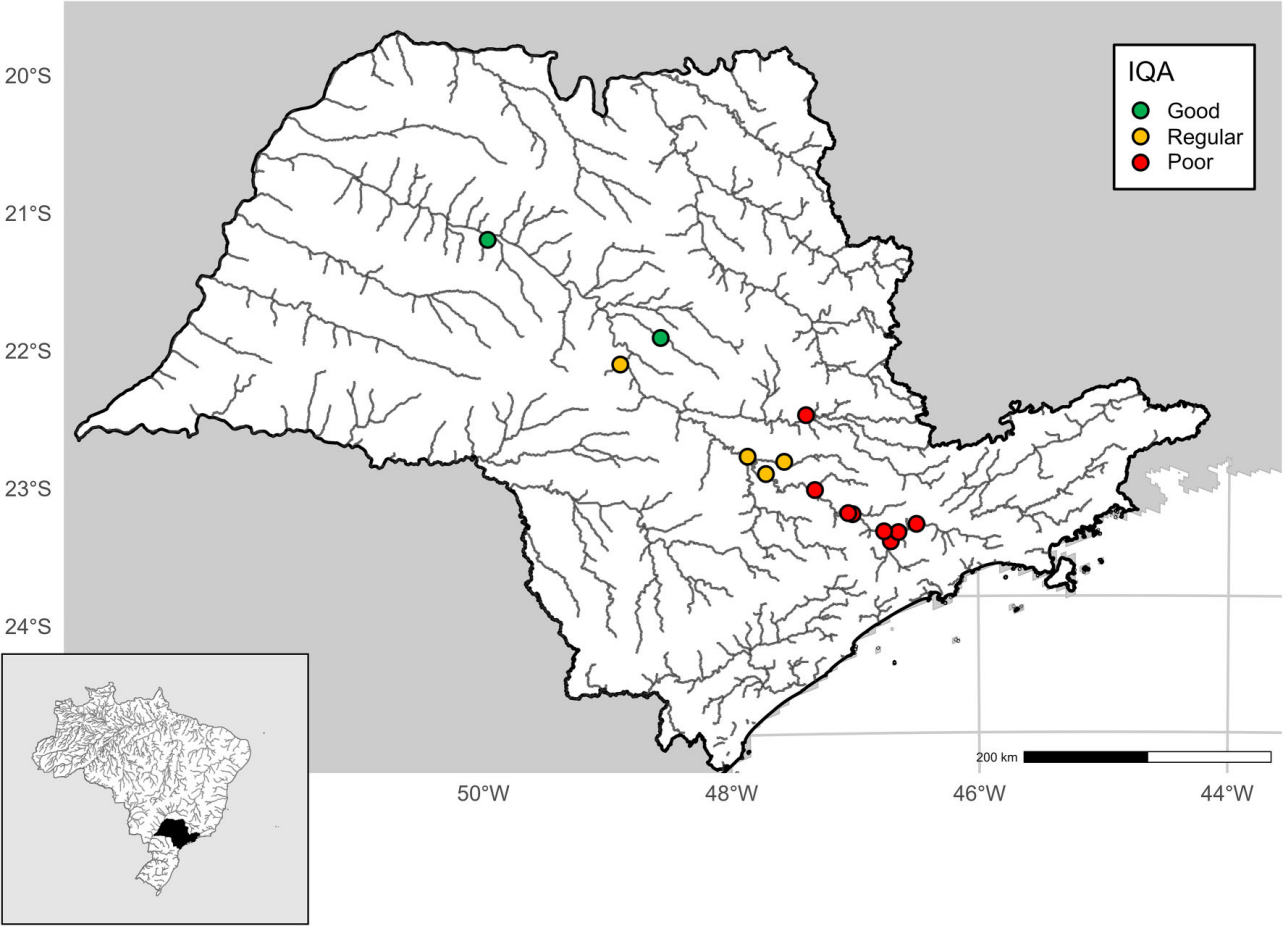
Map of the Tietê River showing all sampling sites. The Water Quality Index (WQI) for each water. The sample is shown in colors: good (green), regular (yellow), and poor (red).

A total of 14 sampling sites were established along the Tietê River Basin, encompassing areas with varying Water Quality Indices (WQI) (Fig. 1, Table S1). In total, 23 samples were collected from 2013 to 2014. These sites are part of the Water Quality Monitoring Program of São Paulo State conducted by CETESB (Environmental Company of São Paulo State). Based on physicochemical, biological, and microbiological parameters, CETESB adopts different water quality indices. The WQI used in this study was previously described (Ortiz-Vera et al. 2018) and it is also available on the official CETESB website (Table S1) (https://www.cetesb.sp.gov.br/cetesb/qualidade_ambiental/agua/aguas_interiores).

To assess the spatial environmental heterogeneity, a Principal Component Analysis (PCA) was performed on the log-transformed physicochemical dataset using the prcomp function in R. The resulting principal components (PCs) were used to visualize the sampling sites according to their environmental profiles. The contribution of each physicochemical variable to the principal components was extracted from the PCA rotation matrix. All statistical analyses and graphical visualizations were conducted in R v4.3.2. The PCA biplot was generated using the ggplot2 package (v3.4.4).

### DNA extraction and 16S rRNA gene sequencing

Water samples (5 l) were collected from the 14 sampling sites (Fig. 1) between August 2013 and April 2014. For microbial biomass collection, one liter of water from each sample was sequentially filtered in triplicate through 1.2 μm and 0.2 μm pore-size cellulose ester membrane (Merck Millipore, Darmstadt, Germany). Only the 0.2 μm membranes were retained for downstream DNA extraction. Total genomic DNA was extracted from the filters using the PowerSoil® DNA Isolation Kit (MoBio Laboratories, Inc., USA) according to the manufacturer’s protocol. Prior to extraction, filters were flash-frozen with liquid nitrogen and mechanically macerated to facilitate cell lysis.

The hypervariable V3-V4 region of the bacterial 16S rRNA gene was amplified from the extracted DNA for high-throughput sequencing. Amplification was performed using the primers: forward primer (5′-TCGTCGGCAGCGTCAGATGTGTATAAGAGACAGCCTACGGGNGGCWGCAG-3′), reverse primer (5′-GTCTCGTGGGCTCGGAGATGTGTATAAGAGACAGGACTACHVGGGTATCTAATCC-3′. The PCR conditions consisted of an initial denaturation at 95°C for 3 min, followed by 25 cycles of 95°C for 30 s, 55°C for 30 s, and 72°C for 30 s, with a final extension at 72°C for 5 min. Amplicons were purified using Ampure Beads (Agencourt, USA), and Illumina sequencing adapters and dual-index barcodes were attached in a second PCR step using the Nextera XT DNA Sample Preparation Kit (Illumina, USA). This indexing PCR comprised eight cycles under the same thermal conditions as the first amplification, followed by a second purification with Ampure Beads. The final libraries were quantified, normalized, and pooled in equimolar ratios. The pooled library was denatured with NaOH, diluted in hybridization buffer, heat-denatured, and sequenced on an Illumina MiSeq platform (Illumina Inc., San Diego, CA, USA) using a 2 × 250 bp paired-end, following the manufacturer’s guidelines.

### 16S rRNA gene amplicon data processing and bioinformatic analysis

Raw sequencing data (single end reads) were processed using the QIIME2 pipeline (version 2023.5) (Bolyen et al. 2019). A manifest file was first created to import the demultiplexed FASTQ files into a QIIME2 artifact. Sequence quality control, denoising, and Amplicon Sequence Variant (ASV) inference were performed using the DADA2 plugin within QIIME2 (Callahan et al. 2016). Based on the quality profile, sequences were trimmed at the 5′-end (15 base pairs) and truncated at the 240 base-pair position to maintain high-quality data. Taxonomic assignment of the representative ASVs was carried out using a pre-trained Naïve Bayes classifier. The classifier was based on the SILVA 138 SSU rRNA reference database (Quast et al. 2013), targeting the V3-V4 hypervariable region. The resulting taxonomy was used to create a bar plot visualization of community composition across samples.

Phylogenetic analysis was conducted to enable phylogenetic diversity metrics. Representative ASV sequences were aligned with MAFFT and used to construct a phylogenetic tree with FastTree. The tree was subsequently rooted at its midpoint to produce a rooted phylogenetic tree for downstream analysis. Prior to diversity analysis, samples were rarefied to ensure even sampling depth and to avoid bias from unequal sequencing effort. A rarefaction depth of 100 000 sequences per sample was chosen.

### Statistical and ecological data analysis

All statistical analyses and data visualizations were performed in R. To assess differences in taxonomic composition and alpha-diversity indices among water quality groups, boxplots were generated using the ggplot2 package. Alpha-diversity metrics, including Shannon, Simpson, Richness, and Pielou’s evenness indices, were calculated using the vegan package. Significant differences among groups were evaluated using analysis of variance (ANOVA), followed by a post-hoc Tukey’s Honest Significant Difference (HSD) test, implemented with the TukeyHSD function from the base R stats package.

The relative abundance of dominant bacterial phyla was visualized using a circos plot, generated with the circlize package (v0.4.16) to illustrate the distribution and connectivity of major taxonomic groups across water quality categories. A Principal Component Analysis (PCA) was employed to visualize the overall variation in bacterial community composition (beta-diversity) based on Hellinger-transformed ASV abundance data. The analysis was performed using the prcomp function. Prior to analysis, environmental variables were normalized to the 0–1 range (min-max normalization) to allow direct comparison. The relationship between bacterial community structure and environmental parameters was tested using a Permutational Multivariate analysis of variance (PERMANOVA) with 999 permutations, implemented via the adonis2 function in the vegan package. Both a global model (all variables) and individual models for each environmental variable were fitted using Bray–Curtis dissimilarities.

Furthermore, a Mantel test was conducted to assess the correlation between the bacterial community dissimilarity matrix (Bray–Curtis) and the normalized environmental variable matrix (Euclidean distance), using the mantel function in the vegan package with 999 permutations. To identify which environmental variables significantly associated with bacterial community structure, the envfit function (vegan package) was used to fit environmental vectors to the PCA ordination. Only vectors with a permutation test *P*-value < 0.05 were considered significant and displayed on the final PCA biplot, which was annotated using the ggplot2 package.

### Analysis of microbial community assembly processes

To quantify the relative importance of ecological processes governing bacterial community assembly, we utilized the iCAMP framework (Ning et al. 2020). This phylogenetic-based method partitions the relative contributions of five key processes: homogeneous selection (HoS), heterogeneous selection (HeS), homogenizing dispersal (HD), dispersal limitation (DL), and drift (DR).

The analysis was conducted using the rooted phylogenetic tree of ASVs and the rarefied ASV abundance table. The phylogenetic tree was first divided into phylogenetic bins using a threshold of 0.2 for the maximum phylogenetic distance (ds) and a minimum bin size limit of 24 taxa. For each bin, the weighted beta Net Relatedness Index (βNRI) and the modified Raup–Crick metric (RCbray; based on Bray–Curtis dissimilarity, abundance-weighted) was calculated across all pairwise sample comparisons (n = 999 permutations). These metrics were used to infer the dominant assembly process for each bin according to established criteria: βNRI ←1.96 indicates HoS; βNRI > 1.96 indicates HeS; |βNRI| ≤ 1.96 and RC > 0.95 indicates DL; |βNRI| ≤ 1.96 and RC ←0.95 indicates HD; and |βNRI| ≤ 1.96 and |RC| ≤ 0.95 indicates DR.

The relative contribution of each process was calculated as the proportion of bins dominated by that process, weighted by their relative taxonomic abundance. To assess the robustness of the results, bootstrapping (999 permutations) was used to compare process importance across different water quality groups. Additionally, complementary approaches were applied, including the Neutral Community Model to estimate the percentage of neutrally distributed taxa, and the Normalized Stochasticity Ratio (NST) to quantify community assembly stochasticity from both taxonomic (tNST) and phylogenetic (pNST) perspectives. To visualize the phylogenetic distribution of the dominant assembly processes across the bacterial tree, the results were imported and graphically represented using the Interactive Tree Of Life (iTOL) online platform (Letunic and Bork 2021).

## Results

### Environmental gradients and water quality classification

**Figure 2:**
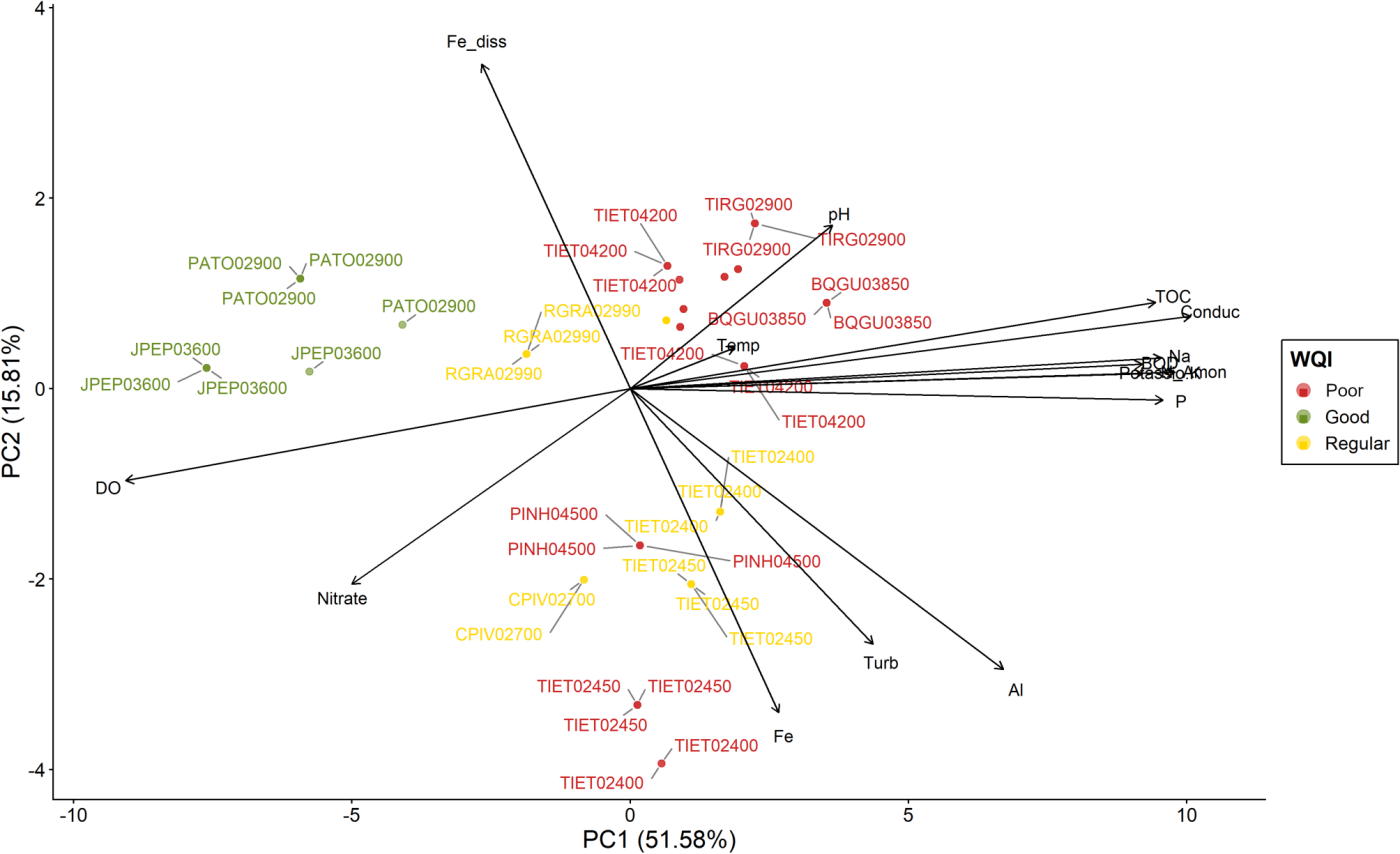
Principal component analysis (PCA) ordination of physicochemical parameters across sampling sites in the Tietê River basin. Symbols represent sampling sites colored according to their Water Quality Index (WQI) classification. Vectors show the direction and strength of significant (*P* < 0.05) environmental variables correlated with the ordination axes. Dissolved oxygen (DO), nitrate, dissolved iron measured after 0.45 µm filtration (Fe_diss), turbidity (Turb), aluminum (Al), total phosphorus (Pt), total iron measured in unfiltered samples (Fe), ammoniacal nitrogen (N_Amon), pH, sodium (Na), potassium (K), temperature (Temp), total organic carbon (TOC) and biochemical oxygen demand (BOD).

Principal Component Analysis (PCA) of physicochemical parameters revealed distinct environmental gradients across the sampling sites, with the first two axes explaining 67.39% of the total variance (PC1: 51.58%; PC2: 15.81%) (Fig. 2). The ordination showed a clear separation of sites as having good Water Quality Index (WQI), specifically PATO02900 and JPEP03600, which formed a distinct cluster. In contrast, sites classified with poor and regular WQI exhibited a broader, although still clustered, distribution along the environmental gradients.

Notably, samples from the TIET02400 and TIET02450 sites (across both 2013 and 2014 sampling years) formed a tightly grouped cluster within the poor/regular WQI category (Fig. 2). Site distribution was significantly correlated with specific environmental variables. Dissolved oxygen (DO) and nitrate concentration were the primary drivers associated with the cluster of good WQI sites. Conversely, the distinct cluster formed by sites TIET02400 and TIET02450 was strongly associated with higher levels of Iron (Fe), turbidity, and Aluminum (Al). The remaining physicochemical parameters were correlated with the broader groups of sites characterized by poor and regular water quality.

### Alpha-diversity patterns across water quality gradients

**Figure 3:**
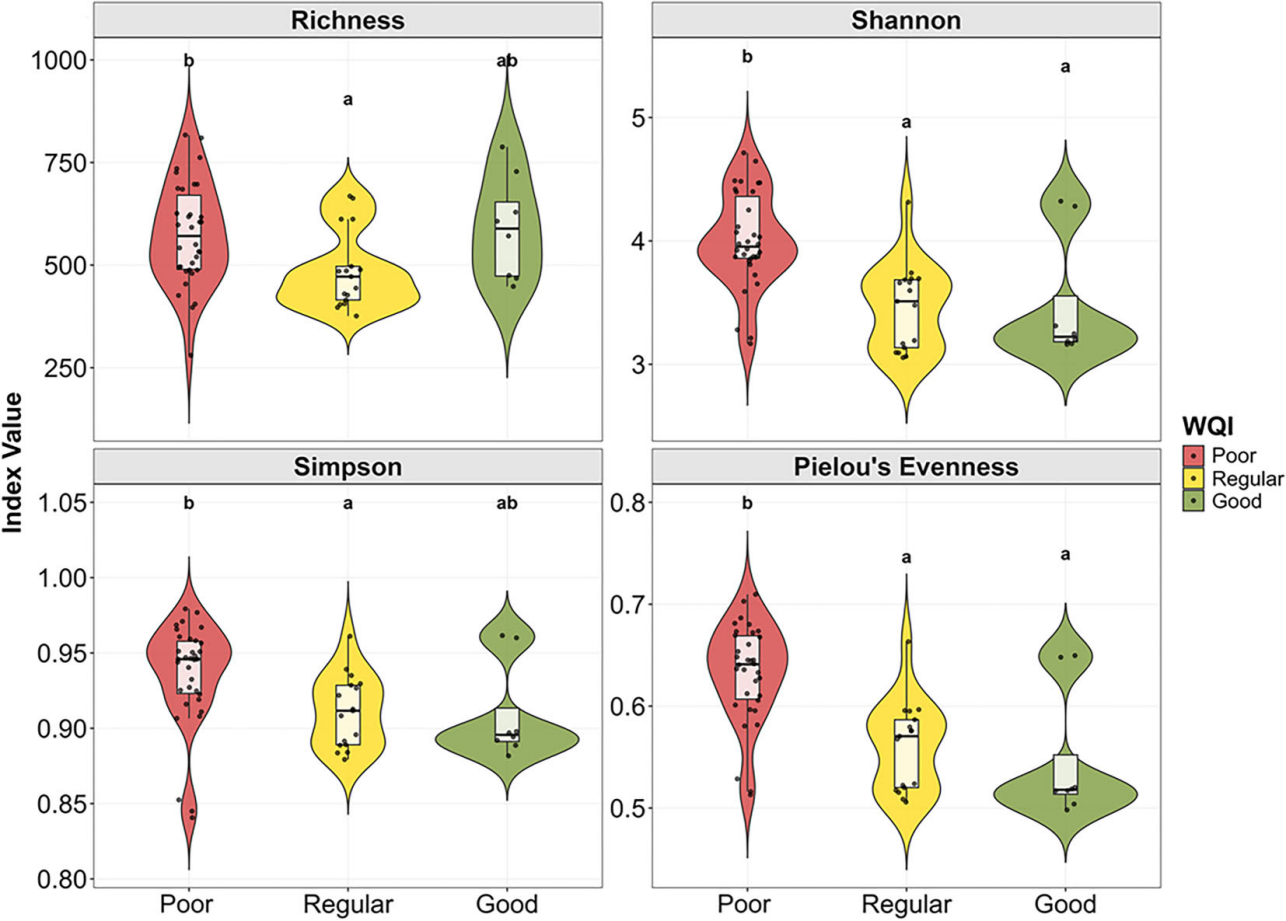
Violin plots illustrating the distribution of four alpha-diversity indices (Richness, Shannon, Simpson, and Pielou’s Evenness) across sampling sites categorized by Water Quality Index (WQI: Poor, Regular, Good). Each panel shows the variability and central tendency of diversity metrics, with points representing individual observations and embedded boxplots summarizing median and interquartile ranges. Significant differences among WQI groups were assessed by one-way ANOVA (df = 2, 56) followed by Tukey’s HSD post hoc test: Richness (F = 3.76, *P* = 0.029), Shannon (F = 13.70, *P* < 0.001), Simpson (F = 4.64, *P* = 0.014), and Pielou’s Evenness (F = 17.61, *P* < 0.001). Different letters above the groups indicate statistically significant differences (*P* < 0.05). Groups sharing the same letter are not significantly different from each other.

Analysis of the ASV table revealed distinct alpha-diversity patterns across WQI categories (Fig. 3). Contrary to initial expectations, sites classified with a ‘Poor’ WQI exhibited significantly higher richness, Shannon and Simpson diversity indices, as well as Pielou’s evenness compared to sites classified as ‘Regular’ or ‘Good’ WQI. This pattern suggests lower taxonomic dominance and more equitable species distributions in highly polluted sites. In contrast, ‘Good’ WQI sites, despite harboring lower overall diversity, were characterized by strong numerical dominance of a few specialized taxa (e.g. *Comamonadaceae* and *Spirosomaceae*), as reflected by their lower Simpson and Pielou indices. No significant differences in alpha-diversity indices were detected between sites classified as ‘Regular’ and ‘Good’.

### Taxonomic shifts in bacterial community composition

**Figure 4:**
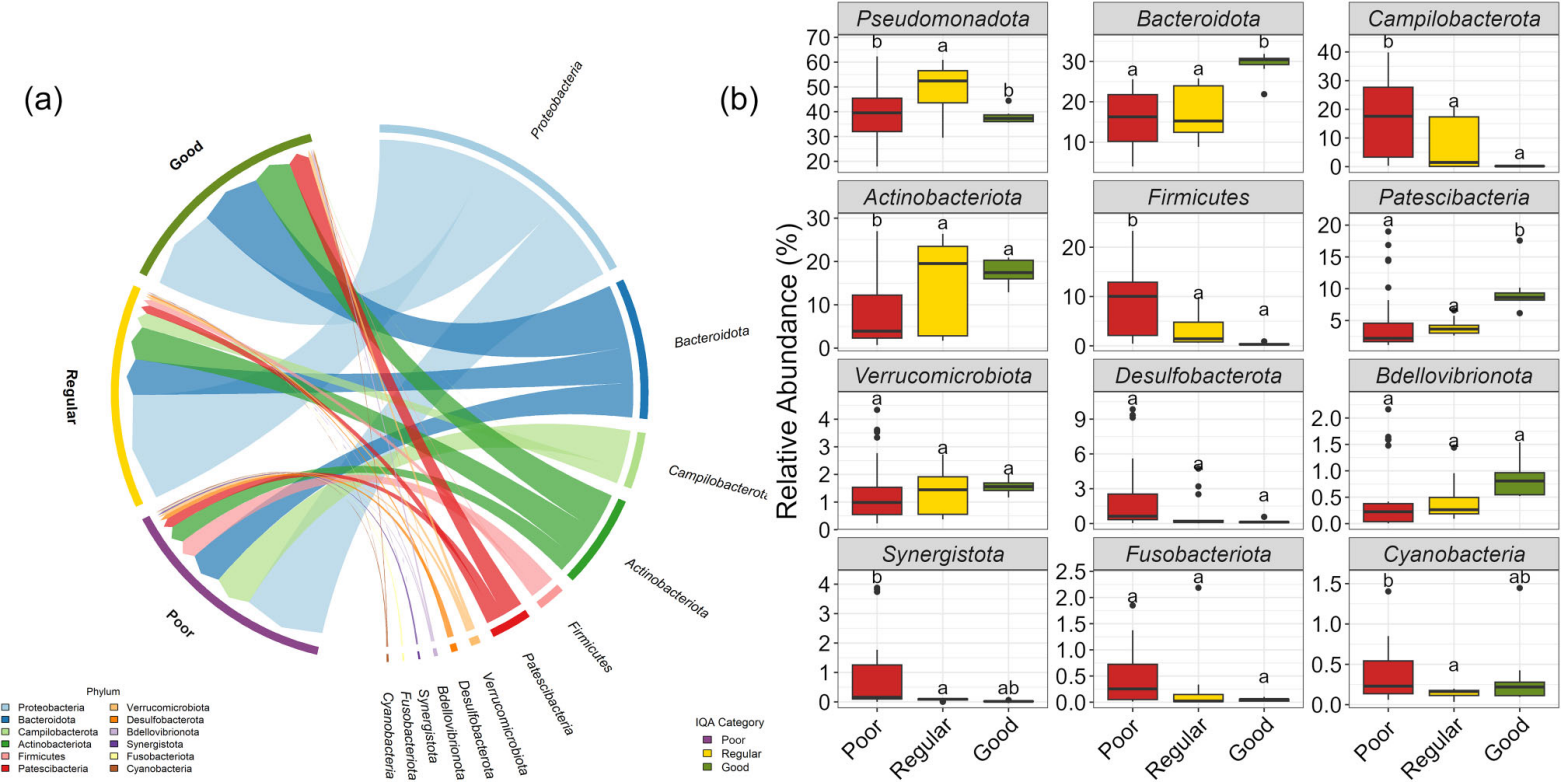
Taxonomic composition and relative abundance of bacterial phyla in the Tietê River. (a) Chord diagram showing the distribution and associations of major bacterial phyla across sampling sites. (b) Relative abundance (%) of the 12 most abundant bacterial phyla across the sampling locations. Significant differences among WQI groups were assessed by one-way ANOVA (df = 2, 56) followed by Tukey’s HSD post hoc test: Actinobacteriota (F = 5.79, *P* = 0.005), Bacteroidota (F = 14.80, *P* < 0.001), Campilobacterota (F = 10.56, *P* < 0.001), Cyanobacteria (F = 3.49, *P* = 0.037), Desulfobacterota (F = 3.20, *P* = 0.048), Firmicutes (F = 13.51, *P* < 0.001), Fusobacteriota (F = 3.49, *P* = 0.037), Patescibacteria (F = 6.33, *P* = 0.003), Proteobacteria (F = 9.33, *P* < 0.001), Synergistota (F = 4.31, *P* = 0.018). Bdellovibrionota and Verrucomicrobiota showed no significant differences (*P* > 0.05). Different letters above boxplots indicate statistically significant differences.

Taxonomic classification at the phylum level revealed a bacterial community dominated by Pseudomonadota (Proteobacteria; 41.29%), followed by Bacteroidota (17.98%), Campylobacterota (12.29%), and Actinobacteriota (11.62%) across all samples (Fig. 4a). Several phyla, including Cyanobacteria (0.29%), Fusobacteriota (0.34%), Synergistota (0.45%), and Bdellovibrionota (0.48%), were classified as components of the rare biosphere. Although a standardized abundance threshold for defining the rare biosphere remains.

The relative abundance of the dominant phyla varied significantly along the WQI gradient (Fig. 4b). The phyla Campylobacterota and Firmicutes were significantly more abundant in ‘Poor’ WQI sites (17.83% and 8.96%, respectively) compared to ‘Regular’ (6.94% and 2.75%) and ‘Good’ (0.17% and 0.38%) sites. In contrast, Actinobacteria were more abundant in ‘Good’ and ‘Regular’ WQI sites (17.63% and 15.41%, respectively) than in ‘Poor’ sites (8.30%). Bacteroidota exhibited a significantly higher relative abundance in ‘Good’ WQI sites (29.26%) than in ‘Regular’ (16.94%) or ‘Poor’ (15.85%) sites.

**Figure 5:**
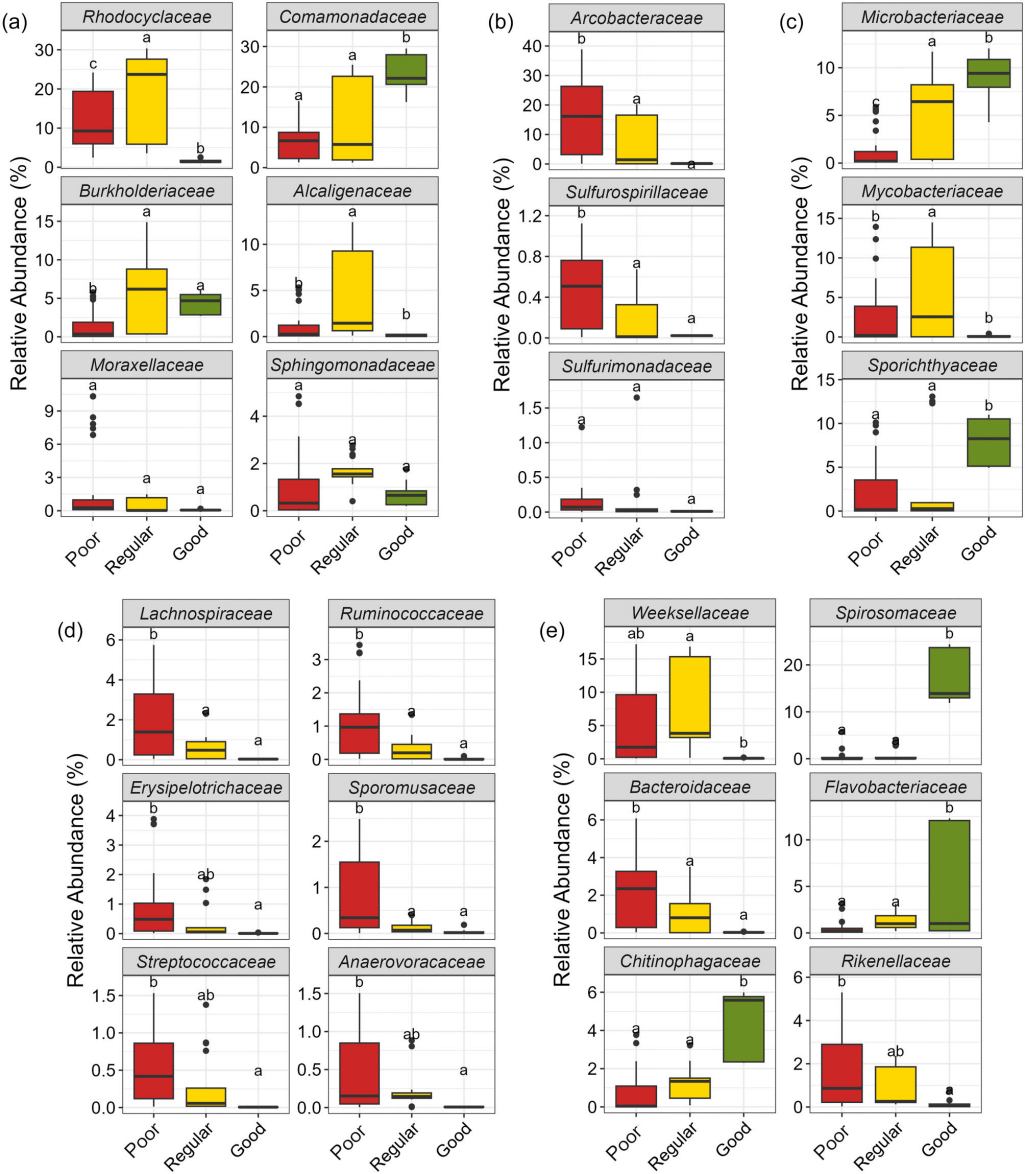
Relative abundance of the most representative bacterial families in the Tietê River. Boxplots show the distribution of the most abundant bacterial families across sampling sites distributed by phylum level. Phylum: (a) Pseudomonadota, (b) Campilobacterota, (c) Actinobacteriota, (d) Firmicutes, and (e) Bacteroidota. Significant differences among WQI groups were assessed by one-way ANOVA (df = 2, 56) followed by Tukey’s HSD post hoc test. F-statistics for families with significant differences: Arcobacteraceae (F = 10.38), Alcaligenaceae (F = 12.53), Burkholderiaceae (F = 14.28), Chitinophagaceae (F = 40.07), Comamonadaceae (F = 21.07), Flavobacteriaceae (F = 12.47), Lachnospiraceae (F = 9.24), Microbacteriaceae (F = 28.96), Mycobacteriaceae (F = 6.07), Rhodocyclaceae (F = 11.90), Ruminococcaceae (F = 9.74), Spirosomaceae (F = 188.25), Sporichthyaceae (F = 8.33), Sporomusaceae (F = 7.29); all *P* < 0.05. Different letters above the boxes indicate statistically significant differences.

Analysis at the family level identified Rhodocyclaceae (12.41%), Arcobacteraceae (11.71%), and Comamonadaceae (9.83%) as the most abundant families across the Tietê River, although their distributions varied markedly along the WQI (Fig. 5). Within Pseudomonadota (Fig. 5a), the relative abundance of Rhodocyclaceae was significantly higher in ‘Regular’ (18.29%) and ‘Poor’ (12.00%) WQI sites compared to ‘Good’ sites (1.64%). A similar pattern was observed for Alcaligenaceae, which was least abundant in ‘Good’ sites (0.14%) and increased in ‘Poor’ (1.17%) and ‘Regular’ (5.20%) sites. In contrast, Comamonadaceae was dominant in ‘Good’ WQI sites (23.22%) compared to both ‘Regular’ (10.53%) and ‘Poor’ (6.33%) sites. The phylum Campylobacterota, significantly enriched in ‘Poor’ WQI sites, was primarily represented by the families Arcobacteraceae and Sulfurospirillaceae (Fig. 5b). Conversely, Actinobacteriota, which was less abundant in ‘Poor’ sites, was mainly represented by Microbacteriaceae and Sporichthyaceae, both significantly more abundant in ‘Good’ WQI (JPEP03600 and PATO02900) environments (Fig. 5c).

The increased relative abundance of Firmicutes in sites classified as ‘Poor’ WQI was associated with the enrichment of multiple families, including Lachnospiraceae, Ruminococcaceae, Erysipelotrichaceae, Sporomusaceae, Streptococcaceae, and Anaerovoracaceae, all significantly more abundant under highly polluted conditions (Fig. 5d). These taxa are commonly associated with anaerobic or facultatively anaerobic lifestyles and are frequently reported in environments characterized by elevated organic loads. Within the phylum Bacteroidota, family-level responses varied markedly along the water quality gradient (Fig. 5e). Weeksellaceae and Bacteroidaceae were enriched in ‘Poor’ and ‘Regular’ WQI sites, reaching relative abundances of 4.81% and 7.28% (Weeksellaceae) and 2.29% and 1.02% (Bacteroidaceae), respectively, whereas both families were rare in ‘Good’ sites (≤0.08%). In contrast, Spirosomaceae, Flavobacteriaceae, and Chitinophagaceae were significantly more abundant in ‘Good’ WQI sites (17.12%, 4.90%, and 4.47%, respectively) compared to ‘Poor’ (0.34%, 0.47%, and 0.59%) and ‘Regular’ (0.65%, 1.31%, and 1.24%) sites. These patterns are consistent with a shift from typical freshwater-associated taxa in better-preserved stretches toward pollution-tolerant and organic matter–associated lineages in degraded river sections.

### Distinct ecological processes govern bacterial community assembly across water quality gradients

Collectively, the iCAMP results reveal a clear ecological transition along the pollution gradient. Communities inhabiting Good WQI stretches were predominantly assembled through homogeneous selection, indicating strong deterministic environmental filtering. In contrast, Poor WQI stretches exhibited a substantial increase in drift and dispersal limitation, together accounting for more than half of total assembly processes. These results indicate a shift from deterministic to stochastic-dominated community assembly under severe anthropogenic disturbance.

**Figure 6:**
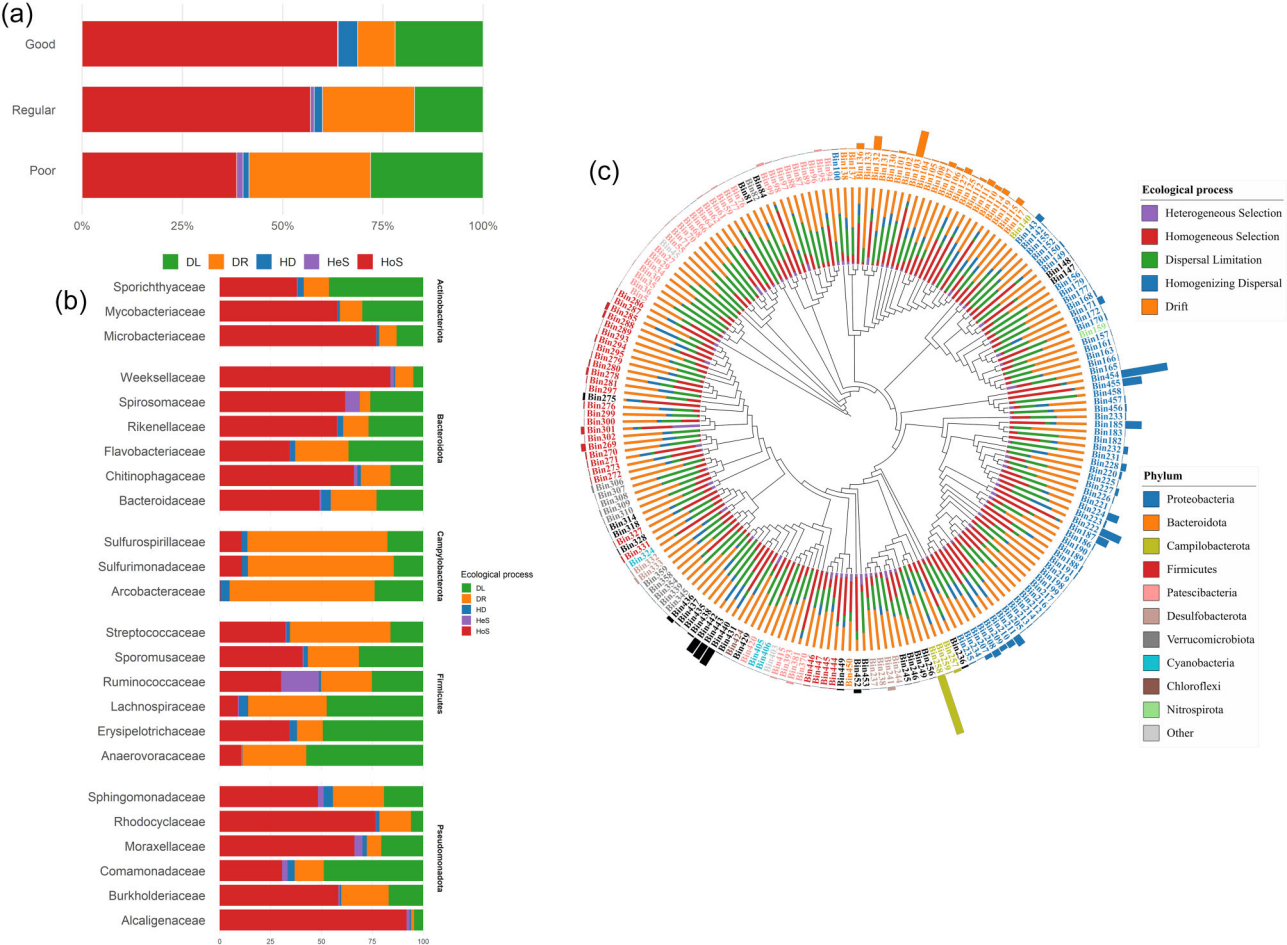
Analysis of ecological community assembly processes along the Tietê River basin using the iCAMP framework. (a) Relative contributions of five ecological processes—homogeneous selection (HoS), heterogeneous selection (HeS), homogenizing dispersal (HD), dispersal limitation (DL), and drift (DR)—across sites grouped by Water Quality Index (WQI). (b) Dominant ecological processes governing the assembly of the most abundant bacterial families within each major phylum. (c) Phylogenetic distribution of the 200 most abundant phylogenetic bins across the basin. The inner ring illustrates the dominant ecological process for each bin, while the outer ring displays the relative abundance of each bin.

Although ecological processes varied among phylogenetic bins (Fig. 6b), consistent patterns emerged across major bacterial groups. Homogeneous selection predominated among abundant freshwater-associated lineages such as Alcaligenaceae, Rhodocyclaceae, Microbacteriaceae, and Weeksellaceae. In contrast, Campylobacterota were assembled predominantly through drift, whereas several Firmicutes and Bacteroidota families exhibited higher contributions of dispersal limitation, indicating that different bacterial groups responded differently to increasing anthropogenic disturbance. Detailed lineage-specific contributions are presented in Fig. 6b.

Among Pseudomonadota, homogeneous selection predominated in most abundant freshwater-associated families, whereas some lineages (e.g. Comamonadaceae and Sphingomonadaceae) exhibited greater influence of dispersal limitation and drift. Campylobacterota were assembled predominantly through drift, whereas Actinobacteriota remained largely governed by homogeneous selection, although some families showed greater influence of dispersal limitation. Firmicutes and Bacteroidota displayed more heterogeneous assembly patterns, with dispersal limitation contributing substantially to several families, indicating greater ecological variability under polluted conditions.

When evaluating the distribution of ecological processes of the most abundant bins along the Tietê River (Fig. 6c), it is possible to verify that even within the evaluated phyla, ecological processes vary drastically within specific bins. Although the most abundant families of the Pseudomonadota phylum exhibit ecological domains governed by homogeneous selection, it can be observed that the most abundant bins indeed present higher values for HoS. However, the rarer bins within the phylum exhibit greater ecological contributions determined by drift. It is also worth highlighting bin 258, related to the Campylobacterota phylum, which is the most abundant bin (considering the relative abundance across the entire Tietê River basin) and shows dominance by drift, followed by heterogeneous selection, probably being the primary contributor to the drift associated with this phylum.

## Discussion

### Community structures in impacted environments

Analysis of diversity indices along the Tietê River revealed that stretches classified as Poor WQI harbored bacterioplankton communities with significantly higher richness, diversity (Shannon and Simpson), and evenness (Pielou) than ‘Regular’ and ‘Good’ stretches (Fig. 3). This pattern contrasts with classical expectations that link anthropogenic disturbance to taxonomic simplification and biodiversity loss (Sattraburut et al. 2025). Rather than indicating ecosystem recovery or ecological stability, these results suggest that chronic, multifactorial, pollution, primarily driven by sustained domestic and industrial effluent inputs, promotes a reorganization of microbial community structure. Specifically, communities appear to shift from assemblages dominated by a limited number of competitive specialists to more taxonomically diverse, yet compositionally distinct, assemblages dominated by opportunistic taxa.

The higher evenness and lower taxonomic dominance observed in ‘Poor’ WQI sites (Fig. 3) may initially appear inconsistent with the predominance of r-strategists in these environments. This apparent paradox is resolved by distinguishing taxonomic from functional dominance. In ‘Good’ WQI stretches, a limited number of well-adapted, oligotrophic specialists exert strong numerical dominance, resulting in low evenness and high taxonomic concentration. In contrast, ‘Poor’ WQI sites are characterized by a phylogenetically diverse assemblage of pollution-tolerant taxa, including members of Firmicutes, Campylobacterota, and Bacteroidota, that occupy distinct but partially overlapping ecological niches associated with differences in organic carbon sources, redox gradients, or metal concentrations. Thus, the high evenness observed under chronic pollution reflects the coexistence of multiple opportunistic lineages, rather than the absence of environmental filtering. Selection appears to favor shared functional traits associated with disturbance tolerance and rapid resource exploitation rather than specific taxonomic groups.

The increased richness observed in Poor WQI stretches is consistent with the mass-effects paradigm of metacommunity theory, in which high immigration rates continuously introduce taxa from surrounding habitats faster than local environmental filtering can remove them (Leibold et al. 2004). Along the Tietê River, untreated sewage, wastewater treatment plant effluents, urban runoff, and tributary inflows likely represent major regional sources of microbial species. Consequently, local diversity may become partially decoupled from environmental suitability, generating highly diverse communities despite severe environmental degradation

Thus, several non-mutually exclusive ecological mechanisms may contribute to this pattern. First, the continuous discharge of untreated or partially treated effluents represents a persistent external inoculum, introducing large numbers of allochthonous micro-organisms, particularly taxa of fecal and intestinal origin, such as Firmicutes and Campylobacterota, which artificially inflate local richness estimates (Hamdhani et al. 2020). Second, the chemical complexity of pollution inputs (including domestic sewage, heavy metals, and agrochemicals) likely increases environmental heterogeneity, promoting the formation of multiple microhabitats and ecological micro niches. This heterogeneity can facilitate the coexistence of metabolically diverse taxa (e.g. denitrifiers, sulfate reducers, ammonia oxidizers, and xenobiotic-tolerant micro-organisms), thereby reducing direct competitive exclusion and supporting higher local diversity, as reported for other chemically stressed aquatic ecosystems (Qiao et al. 2024).

The enrichment of fecal-associated bacterial groups further supports the occurrence of source–sink dynamics. Under this framework, wastewater treatment plants, sewer overflows, and urban drainage systems act as regional source habitats that continuously export micro-organisms into the river. Many of these taxa may persist locally despite suboptimal environmental conditions because immigration rates exceed local extinction rates. Consequently, community composition in polluted stretches may reflect not only local environmental selection but also the sustained influence of upstream anthropogenic sources. Because immigration rates and source contributions were not directly quantified in this study, the relative importance of mass effects versus local species sorting remains an inference based on community composition patterns and known anthropogenic inputs.

Finally, these patterns are consistent with a shift in dominant life-history strategies along the r/K continuum. In stretches with Good WQI, relatively stable physicochemical conditions (e.g. higher dissolved oxygen and lower organic loads) favor K-strategists, specialized, slower-growing, and competitively efficient taxa such as Comamonadaceae and Spirosomaceae, that can dominate community structure and limit diversity. In contrast, highly disturbed and resource-rich Poor WQI stretches favor r-strategists, which are fast-growing, stress-tolerant generalists, including many Firmicutes and members of the Arcobacteraceae. Importantly, the elevated diversity observed under these conditions likely reflects a state of chronic disturbance rather than ecological stability, characterized by opportunistic colonization rather than persistent, functionally cohesive communities (Bouchali et al. 2022, Li et al. 2025).

### Taxonomic signatures of pollution and the suppression of self-purification

Family-level analyses revealed clear taxonomic signatures associated with the degree of anthropogenic impact along the Tietê River (Fig. 5). The pronounced enrichment of Campylobacterota, particularly Arcobacteraceae, and several Firmicutes families in Poor WQI stretches (Fig. 4) is strongly consistent with the physicochemical gradients identified by the PCA. These sites were characterized by reduced dissolved oxygen and elevated concentrations of ammoniacal nitrogen, total phosphorus, biochemical oxygen demand, and turbidity (Fig. 2), conditions typically associated with intense organic pollution and oxygen depletion. Such conditions favor anaerobic or microaerophilic taxa capable of exploiting reduced nitrogen compounds and fermentative substrates. The enrichment of Lachnospiraceae, Ruminococcaceae, Erysipelotrichaceae, and Arcobacteraceae therefore reflect not only anthropogenic contamination but also a shift in the river’s redox regime toward oxygen-limited metabolic processes.

These lineages, typically associated with intestinal microbiomes and reducing environments, not only reflect the source of the impact but also signal a fundamental change in the river’s redox regime, from an aerated system to one dominated by fermentative and sulfur-reducing processes.

Additionally, a marked decline was observed in taxa typically associated with oligotrophic and well-oxygenated freshwater environments. Reduced abundances of Spirosomaceae and Flavobacteriaceae (Bacteroidota), along with Comamonadaceae (Pseudomonadota), in polluted stretches (Figs. 4 and 5) suggest the loss of microbial groups involved in the aerobic degradation of complex polysaccharides and recalcitrant organic compounds (Seveso et al. 2024, Sun et al. 2026). Their replacement by fermentative and reductive communities implies a functional shift that may impair the river’s self-purification capacity, favoring incomplete organic matter degradation and the accumulation of reduced metabolic end products, including organic acids, ammonia, and hydrogen sulfide.

The consistent enrichment of Actinobacteriota and Bacteroidota across all ‘Good’ and ‘Regular’ WQI sites (Fig. 4b) reflects positive environmental selection under less impacted conditions. Within Actinobacteriota, Microbacteriaceae and Sporichthyaceae predominated in ‘Good’ sites (Fig. 5c), both families are typical aerobic oligotrophs in freshwater systems (Newton et al. 2011). Within Bacteroidota, Spirosomaceae, Flavobacteriaceae, and Chitinophagaceae drove the pattern (Fig. 5e), lineages recognized as efficient degraders of complex polysaccharides and proteins in oxic environments (Fernández-Gómez et al. 2013). Their consistent exclusion from polluted stretches indicates strong filtering against aerobic, particle-associated lifestyles under hypoxic, organic-rich conditions. Thus, the loss of these groups in degraded sites represents not merely taxonomic turnover, but the suppression of functional guilds central to aerobic organic matter mineralization and self-purification.

Nevertheless, even under Poor WQI conditions, evidence of adaptive microbial responses was observed. The increased abundance of Rhodocyclaceae in Regular and Poor stretches (Fig. 5) suggests selective enrichment of taxa associated with denitrification and aromatic compound degradation (Keller et al. 2018). This pattern indicates that, under extreme nitrogen and organic carbon loading (Fig. 1), the river may retain limited functional potential for contaminant attenuation, albeit within a profoundly altered ecological context.

### Transition in ecological assembly regimes

Community assembly processes along the Tietê River exhibited pronounced spatial variation across the pollution gradient. In Good and Regular WQI stretches, homogeneous selection (HoS) was the dominant assembly mechanism (Fig. 6), consistent with theoretical expectations that stable and predictable environmental conditions promote deterministic community structuring driven by physicochemical constraints such as dissolved oxygen and nutrient availability (Wang et al. 2021, He et al. 2025). The environmental gradients identified in the PCA provide a mechanistic explanation for these assembly patterns. Good WQI sites were primarily associated with elevated dissolved oxygen (F=12.4269, *P* < 0.001 and nitrate concentrations (F=4.9128, *P* < 0.005), whereas Poor WQI stretches were associated with high ammoniacal nitrogen (F=7.0871, *P* < 0.001), iron (F=6.9334, *P* < 0.001), turbidity (F=4.999, *P* < 0.001) and biochemical oxygen demand (F= 1.8372, *P* < 0.05). Elevated oxygen concentrations likely induce strong deterministic selection favoring aerobic freshwater specialists, such as Comamonadaceae and Spirosomaceae. In contrast, severe oxygen depletion combined with high organic loading expands the range of viable disturbance-tolerant strategies, allowing multiple phylogenetically distinct taxa to occupy functionally similar niches. This shift may reduce taxonomic predictability while maintaining strong selection at the functional level.

However, Poor WQI stretches displayed a marked reduction in the relative contribution of HoS (38.5%), accompanied by a pronounced increase in stochastic processes, particularly ecological drift (DR) and dispersal limitation (DL), which together accounted for over 58% of community assembly (Fig. 6). Similar transitions toward stochastic dominance under severe environmental stress have been reported in other degraded aquatic ecosystems, suggesting a broader ecological pattern (Sachs et al. 2022, Liao et al. 2025). In the Tietê River, this shift likely reflects the destabilization of canonical environmental filters, rather than their intensification.

The elevated contribution of drift (30.2%) in eutrophicated stretches indicates that random birth–death processes and demographic fluctuations increasingly govern local population dynamics. Such dynamics are consistent with ecological theory predicting enhanced stochasticity in systems characterized by small, unstable populations under chronic stress (Nguyen et al. 2021). High dispersal limitation between Good and Poor stretches (45.9%) further reflects the spatial isolation of remaining high-quality habitats (Fig. 1), which act as fragmented refugia. Moreover, significant DL among geographically proximate Poor stretches suggests that extreme pollution functions as a post-dispersal environmental filter, preventing successful establishment despite continuous propagule input (Yin et al. 2021, Cubry et al. 2022). Under these conditions, polluted river sections behave as chemically hostile “islands,” supporting communities that are simultaneously diverse, due to repeated inoculation, and fragmented, due to widespread failure of metabolic adaptation.

An alternative, and not mutually exclusive, explanation for the decline in homogeneous selection is that environmental filtering remains strong under extreme pollution but acts primarily on functional traits rather than taxonomic identity. Persistent hypoxia, elevated organic loads, and chemical contamination are likely selected for disturbance-tolerant traits, including anaerobic metabolism, rapid resource acquisition, and stress resistance. Because these traits are shared across phylogenetically distinct taxa, functional convergence may occur without equivalent taxonomic convergence (Wang et al. 2020, Liu et al. 2021). Under this scenario, the increased contribution of drift and dispersal-related processes reflects demographic instability, continuous anthropogenic immigration, and habitat fragmentation, rather than a complete loss of environmental selection.

This fine-scale phylogenetic analysis revealed a critical layer of complexity in assembly mechanisms: ecological processes are not uniform across entire phyla but vary significantly among phylogenetic bins within the same lineage (Fig. 6c). The strong homogeneous selection observed in Alcaligenaceae, Rhodocyclaceae, and Moraxellaceae (Fig. 6b) reflects environmental filtering by stable, high-concentration carbon supplies and consistent redox conditions, characteristic of the organically enriched ‘Regular’ and ‘Poor’ WQI sites where these families were most abundant (Fig. 5a). Homogeneous selection intensifies under predictable substrate availability, and these families are typically associated with aerobic respiration, denitrification, and labile carbon degradation. Their deterministic assembly is therefore a consequence of sustained, spatially consistent anthropogenic forcing, not an intrinsic lineage property. This intraphylum heterogeneity underscores that microbial traits and ecological strategies are phylogenetically conserved at a finer scale than the phylum or even family level. For instance, within Pseudomonadota, while dominant families like Rhodocyclaceae were governed by homogeneous selection, the assembly of rarer bins within the same phylum was predominantly shaped by drift. This pattern suggests that abundance itself is a key trait filtering assembly process. Abundant, widespread taxa (likely generalists or well-adapted specialists) are subject to strong and consistent environmental selection (HoS). In contrast, rare taxa within the same broad lineage may exist as small, vulnerable populations whose dynamics are more susceptible to stochastic birth-death events (DR), reflecting their status as transient or satellite members of the community.

Notably, bin 258 (Campylobacterota), the most abundant bin across the basin, was assembled predominantly through drift. This finding challenges the simple assumptions linking high abundance to deterministic niche optimization. This finding challenges the assumption that high abundance necessarily reflects deterministic niche optimization (Pearce et al. 2020, Wang et al. 2020). Instead, it suggests that the dominance of Arcobacteraceae reflects sustained external inoculation combined with neutral demographic processes, rather than selective advantage per se. Such ‘neutral blooms’ are characteristic of systems where stochastic processes override niche-based assembly and contribute to ecological unpredictability (Pearce et al. 2020).

This transition to a stochasticity-dominated assembly regime has profound implications for ecosystem resilience and functioning. Communities with ecological processes determined mainly by drift and with high dispersal limitation are intrinsically less stable and less predictable in response to future disturbances, representing an altered ecological state and potentially less resilient for the Tietê River (Philippot et al. 2021, Lerch et al. 2023).

The ecological regime governing the rare biosphere provides additional evidence of collapsed resilience in the Tietê River. Theoretical and empirical work has emphasized that rare microbial taxa can play a disproportionate role in ecosystem recovery following disturbance (Bontemps et al. 2024). In the Lascaux Cave system rare taxa exhibited a transition from stochastic to deterministic assembly during post-disturbance attenuation, suggesting that deterministic selection on rare lineages is a mechanistic component of microbial resilience (Bontemps et al. 2024). In the Tietê River, however, stochastic processes (drift and dispersal limitation) remained dominant across rare phylogenetic bins even in severely polluted stretches (Fig. 6c). This persistent stochastic regime indicates that the rare biosphere is functionally locked in a state of chronic disturbance, unable to undergo the deterministically driven reorganization associated with ecological recovery.

Some limitations have been considered when interpreting these results. First, both ecological assembly processes and ecosystem functions were inferred indirectly from phylogenetic turnover, taxonomic composition, and environmental context rather than directly measured. Although iCAMP provides a robust framework for partitioning community assembly mechanisms, it cannot directly quantify dispersal, demographic stochasticity, or environmental selection, and its inferences may be influenced by continuous anthropogenic inputs that generate source–sink dynamics and mass effects. Similarly, interpretations regarding ecosystem functioning, including self-purification capacity, reductive metabolism, and biogeochemical transformations, are based on the known ecological attributes of dominant taxa and should therefore be considered functional inferences rather than direct measurements. Consequently, the ecological mechanisms proposed here should be viewed as plausible explanations supported by observed community patterns, requiring further validation through approaches such as metagenomics, metatranscriptomics, metabolomics, or direct measurements of ecosystem processes. Partial functional support for these inferences, however, is provided by a complementary metagenomics study conducted on the Tietê River during the same sampling period (Martinez et al. 2026). Using shotgun metagenomics across a comparable WQI gradient, including overlapping sampling sites, that study found direct functional evidence consistent with the metabolic shift inferred here, including genes associated with fermentation were enriched at the most polluted site, while genes underpinning aerobic carbon metabolism (CO₂ fixation, glycolysis, and the TCA cycle) predominated at oligotrophic, higher-quality sites. Nitrogen cycling genes involved in denitrification and nitrate reduction were also most abundant at polluted sites, whereas nitrification-associated genes were undetectable throughout the basin. These convergent taxonomic and functional patterns strengthen the interpretation that chronic pollution drives a shift from aerobic, self-purifying metabolic regimes toward anaerobic and fermentative pathways in the Tietê River.

### The tietê river as a model of forced ecological transition

Collectively, our results demonstrate that chronic anthropogenic pollution in the Tietê River induces a fundamental transition in the ecological regime governing its aquatic microbiome. Along with the water quality gradient, communities shift from deterministically structured assemblages under Good WQI conditions to stochasticity-dominated assemblages in severely polluted stretches. This transition is accompanied by the replacement of sensitive freshwater specialists (K-strategists) by diverse but unstable assemblages of opportunistic generalists (r-strategists).

Communities assembled primarily through stochastic processes are, by definition, less predictable and less resilient to further disturbance (Leonov 2023). Accordingly, the high alpha diversity observed in the most polluted stretches (Fig. 3) may characterize a community dominated by opportunistic micro-organisms, but devoid of the stable functional redundancy and complex biotic interactions that confer resistance to disturbances in less impacted ecosystems (Philippot et al. 2021). Alterations in the microbial diversity of the Tietê River may disrupt self-purification processes. The decrease in specific bacterial groups involved in aerobic degradation, such as members of the Comamonadaceae and Spirosomaceae families, combined with the increase in reductive and fermentative metabolic pathways, are consistent with reduced self-purification capacity. The concurrent loss of taxa involved in aerobic organic matter degradation and the rise of fermentative and reductive pathways suggest impaired self-purification capacity, increasing the likelihood of accumulating toxic intermediate and greenhouse gases such as methane and nitrous oxide within the basin (Beaulieu et al. 2019).

Several limitations should be considered when interpreting these results. First, this study is based on an observational survey and therefore identifies associations rather than direct causal relationships between environmental conditions and microbial community patterns. Consequently, the proposed ecological mechanisms should be viewed as plausible interpretations supported by observed patterns rather than definitive demonstrations of causality. In addition, unmeasured environmental variables, hydrological dynamics, seasonal variation, pollutant mixtures, and historical contingencies may have contributed to the observed community responses. A further limitation is that samples were collected between 2013 and 2014, and the taxonomic composition reported here should therefore be considered representative of the study period rather than the current state of the Tietê River. Nevertheless, the pronounced pollution gradient provided a valuable framework for investigating broader ecological responses to anthropogenic disturbance, although contemporary surveys are needed to assess the persistence of these patterns under present-day conditions.

Contrary to our initial hypothesis, our results suggest that deterministic environmental filtering became relatively less important at the taxonomic level. This inversion underscores that severe anthropogenic stress can destabilize, rather than reinforce, classical niche-based assembly rules. We therefore propose that the metropolitan stretches of the Tietê River represent an alternative ecological state imposed by chronic pollution, characterized by high taxonomic diversity sustained by continuous inoculation, strong selection for disturbance-tolerant functional traits, increased influence of stochastic assembly processes, and reduced ecosystem resilience. Effective management and restoration efforts should thus aim not only to reduce contaminant loads but also to re-establish environmental conditions, even considering the presence of urban and rural areas, that enable deterministic selection and metacommunity connectivity, which are essential for restoring microbial-mediated ecosystem functions.

## Supplementary Material

fiag083_Supplemental_File

## Data Availability

The datasets generated during the current study are available in the NCBI repository under project PRJNA1427712.
