# Supplementary material for: Extreme pollution reshapes microbial community assembly in a tropical urban river
Source: FEMS Microbiol Ecol. 2026 Jul 29;102(8):fiag083. doi: 10.1093/femsec/fiag083 (PMC13441173; doi:10.1093/femsec/fiag083)
Supplement: Supplementary file 1 [file fiag083_supplemental_file.docx]

| Sample | Geographical coordinates | Year | WQI | Total Iron  (mg L⁻¹) | Total Phosphorus  (mg L⁻¹) | Ammoniacal Nitrogen  (mg L⁻¹ as N) | Nitrate (mg L⁻¹ as NO₃⁻) | Dissolved Oxygen (DO)  (mg L⁻¹) |
| --- | --- | --- | --- | --- | --- | --- | --- | --- |
| CPIV 02700 | 22.9994 S 47.5311 W | 2013 | Regular | 8,5 | 0,3 | 3 | 1 | 3,2 |
| JPEP 03600 | 22.0733 S 48.4867 W | 2013 | Good | 1,5 | 0,02 | 0,1 | 0,7 | 7,8 |
| PATO 02900 | 21.3214 S 49.8222 W | 2013 | Good | 1,7 | 0,08 | 0,1 | 1,2 | 7,1 |
| PINH 04500 | 23.5939 S 46.6936 W | 2013 | Poor | 1,8 | 2,2 | 17,1 | 0,2 | 1,5 |
| TIET 02400 | 23.0867 S 47.6781 W | 2013 | Poor | 8,1 | 1,7 | 22,1 | 0,1 | 0,8 |
| TIET 02450 | 22.9572 S 47.8206 W | 2013 | Poor | 7,9 | 1,4 | 17,9 | 0,7 | 0,7 |
| TIET 04200 | 23.5197 S 46.7464 W | 2013 | Poor | 3,7 | 2,3 | 13,1 | 0,2 | 0,4 |
| TIRG 02900 | 23.3828 S 47.0294 W | 2013 | Poor | 2,9 | 3,1 | 19,1 | 0,2 | 0,5 |
| BQGU 03850 | 23.4675 S 46.4878 W | 2014 | Poor | 2,6 | 1,2 | 14,2 | 0,5 | 0,07 |
| JPEP 03600 | 22.0733 S 48.4867 W | 2014 | Good | 2,9 | 0,08 | 0,3 | 1,2 | 6,9 |
| JUNA 04900 | 23.2100 S 47.2911 W | 2014 | Poor | 0,9 | 1 | 7 | 0,7 | 0,4 |
| PATO 02900 | 21.3214 S 49.8222 W | 2014 | Good | 2,3 | 0,4 | 0,2 | 1 | 2,4 |
| PINH 04500 | 23.5939 S 46.6936 W | 2014 | Poor | 5,2 | 0,5 | 4,7 | 0,2 | 0,7 |
| RGRA 02990 | 22.2608 S 48.8097 W | 2014 | Regular | 2,5 | 0,8 | 4,9 | 1 | 2,8 |
| TAMT 04900 | 23.5267 S 46.6322 W | 2014 | Poor | 2,3 | 1,2 | 9,9 | 0,2 | 1,1 |
| TATU 04850 | 22.6600 S 47.3525 W | 2014 | Poor | 2 | 1 | 13 | 0,08 | 0,3 |
| TIET 02400 | 23.0867 S 47.6781 W | 2014 | Regular | 7,9 | 1,3 | 3,2 | 2,1 | 1,6 |
| TIET 02450 | 22.9572 S 47.8206 W | 2014 | Regular | 4,2 | 1,3 | 3,2 | 1,8 | 1,8 |
| TIET 04200 | 23.5197 S 46.7464 W | 2014 | Poor | 2,3 | 0,9 | 9,3 | 0,2 | 0,7 |
| TIPI 04900 | 23.3908 S 46.9947 W | 2014 | Poor | 3,1 | 1,3 | 9,8 | 0,2 | 0,3 |
| TIRG 02900 | 23.3828 S 47.0294 W | 2014 | Poor | 3,3 | 1,2 | 12,6 | 0,2 | 1 |

Table S1: Geographic location (sample code and coordinates), sampling year, Water Quality Index (WQI) classification, and physicochemical parameters measured at each sampling site.

| Sample | pH | Potassium  (mg L⁻¹) | Sodium  (mg L⁻¹) | Temperature  (°C) | Turbidity  (NTU) | Total Aluminum  (mg L⁻¹) | Total Organic  Carbon (TOC) (mg L⁻¹) | Conductivity  (µS cm⁻¹) | Biochemical Oxygen  Demand (BOD) (mg L⁻¹) | Dissolved Iron  (mg L⁻¹) |
| --- | --- | --- | --- | --- | --- | --- | --- | --- | --- | --- |
| CPIV 02700 | 7,3 | 10 | 26 | 22 | 23 | 17 | 6,9 | 209 | 34 | 0,4 |
| JPEP 03600 | 7,1 | 1,3 | 1,4 | 20,2 | 14 | 0,1 | 1,5 | 45 | 2 | 0,47 |
| PATO 02900 | 7 | 3,9 | 10 | 21,1 | 3,5 | 0,1 | 2,7 | 95 | 2 | 0,85 |
| PINH 04500 | 6,9 | 10,2 | 41,3 | 21,6 | 25,8 | 0,6 | 38 | 522 | 55 | 0,44 |
| TIET 02400 | 7,4 | 11 | 45 | 20,2 | 17 | 8,5 | 13,7 | 655 | 14 | 0,1 |
| TIET 02450 | 7,4 | 11,2 | 48,2 | 20,2 | 24 | 8 | 10,9 | 552 | 13 | 0,1 |
| TIET 04200 | 7 | 10,7 | 44,4 | 19,3 | 33,9 | 3,9 | 58,8 | 590 | 96 | 0,76 |
| TIRG 02900 | 7,8 | 10 | 42,3 | 21,4 | 18,4 | 1,5 | 46,6 | 646 | 136 | 0,89 |
| BQGU 03850 | 7,8 | 46 | 75,5 | 24,3 | 41,5 | 5,7 | 44,7 | 1014 | 48 | 0,52 |
| JPEP 03600 | 7,2 | 1,9 | 1,3 | 25,6 | 23 | 0,2 | 5,7 | 100 | 2 | 1,21 |
| JUNA 04900 | 7,1 | 18 | 92 | 31 | 47 | 0,5 | 64,6 | 678 | 96 | 0,3 |
| PATO 02900 | 6,6 | 4,3 | 16 | 25,6 | 17 | 0,1 | 10,4 | 90 | 3 | 1,5 |
| PINH 04500 | 6,8 | 5,6 | 13,8 | 25,4 | 101 | 10,1 | 17,4 | 309 | 46 | 0,4 |
| RGRA 02990 | 7,2 | 3,3 | 15,7 | 27,6 | 31 | 0,4 | 12,3 | 230 | 11 | 0,82 |
| TAMT 04900 | 7,2 | 9,5 | 47,8 | 24,3 | 14,5 | 2,1 | 28,4 | 469 | 40 | 0,32 |
| TATU 04850 | 7,2 | 9 | 56 | 24 | 49,1 | 0,4 | 41,9 | 561 | 52 | 0,3 |
| TIET 02400 | 6,7 | 10,1 | 36,9 | 25,5 | 150 | 12,8 | 25,4 | 264,9 | 36 | 0,1 |
| TIET 02450 | 6,8 | 8,1 | 28,2 | 26,8 | 180 | 8,7 | 19,5 | 225 | 33 | 0,1 |
| TIET 04200 | 7,1 | 9,9 | 42,2 | 26,4 | 18,7 | 1,5 | 27,2 | 448 | 31 | 0,97 |
| TIPI 04900 | 7,1 | 9 | 35,8 | 27,7 | 21,4 | 2,1 | 23,9 | 399 | 26 | 1,3 |
| TIRG 02900 | 7,3 | 8,6 | 32,8 | 27,3 | 14,7 | 1,9 | 20,9 | 475 | 23 | 0,5 |
